# Clinicoradiologic correlates of corticolimbic index (CLix) and glial activation differences among neuropathologic subtypes of Alzheimer’s disease

**DOI:** 10.1002/alz.083977

**Published:** 2025-01-09

**Authors:** Melissa E. Murray, Sara R Dunlop

**Affiliations:** ^1^ Mayo Clinic, Jacksonville, FL USA

## Abstract

**Background:**

Selective corticolimbic vulnerability to tau pathology in Alzheimer’s disease (AD) underlies clinicopathologic heterogeneity. The goal of this presentation will be to examine spatial heterogeneity of tangle distribution on a continuum through the utility of the corticolimbic index (CLix).

**Method:**

We will discuss the development of CLix in the Florida Autopsied Multi‐Ethnic (FLAME) cohort, which sought to collapse the spatial distribution of thioflavin‐S tangle counts in AD (n=1361) to assign a continuum: hippocampal sparing with cortical predominance (<10), representative/typical (≥10 to <30), and limbic predominant with cortical sparing (≥30). The relationship with atypical, non‐amnestic clinical presentations will be described. Relevant clinical measures, including age at symptomatic onset and longitudinal decline on mini‐mental state examination (MMSE) will be examined. Digital pathology measures of AD (tau [AT8, GT‐38], amyloid‐β [6F/3D]) and glial activation (astrogliosis: GFAP, activated microglia/macrophage: CD68) will be shown in hippocampus and association cortices (n=60). To further extend these findings to the antemortem space, CLix score will be examined in an independent, autopsied neuroimaging group (n=93).

**Results:**

Lower CLix score correlated with younger age at symptomatic onset (R=0.39, p<0.001), faster MMSE decline (R=0.27, p<0.001), and associated with non‐amnestic clinical presentations (median=13 vs. 21, p<0.001). Lower CLix score correlated with greater hippocampal volume on MRI (R=‐0.45, p<0.001) and higher flortaucipir‐PET uptake (e.g., posterior cingulate‐precuneus cortex: R=‐0.74, p<0.001). Advanced tangle maturity burden (GT‐38) differed across all regions (p<0.001). Hippocampal sparing AD exhibited higher cortical tau pathology and reduced cortical activated microglia/macrophage.

**Conclusions:**

Corticolimbic tangle distribution links with AD clinical features and neuroimaging changes. Reduced activated microglia/macrophage in association cortices of relative hippocampal sparing AD may contribute to higher tau burden. Corticolimbic vulnerability should be considered for personalized therapies targeting immune dysregulation.

